# Cadmium toxicity induced contrasting patterns of concentrations of free sarcosine, specific amino acids and selected microelements in two *Noccaea* species

**DOI:** 10.1371/journal.pone.0177963

**Published:** 2017-05-19

**Authors:** Veronika Zemanová, Milan Pavlík, Daniela Pavlíková

**Affiliations:** 1 Isotope Laboratory, Institute of Experimental Botany, Academy of Sciences of the Czech Republic, Prague, Czech Republic; 2 Department of Agro-Environmental Chemistry and Plant Nutrition, Faculty of Agrobiology, Food and Natural Resources, Czech University of Life Sciences, Prague, Czech Republic; Sun Yat-Sen University, CHINA

## Abstract

Cadmium (Cd) toxicity affects numerous metabolic processes in plants. In the presence of Cd, plants accumulate specific amino acids which may be beneficial to developing Cd tolerance. Our study aimed to characterize the changes in the metabolism of selected free amino acids that are associated with Cd tolerance, and investigate the levels of selected microelements in order to relate these changes to the adaptation strategies of two metallophytes—*Noccaea caerulescens* (Redlschlag, Austria) and *Noccaea praecox* (Mežica, Slovenia). The plants were exposed to Cd contamination (90 mg Cd/kg soil) for 120 days in a pot experiment. Our results showed higher Cd accumulation in *N*. *praecox* compared to *N*. *caerulescens*. Cadmium contamination reduced the zinc and nickel levels in both species and a mixed effect was determined for copper and manganese content. Differences in free amino acid metabolism were observed between the two metallophytes growing under Cd-free and Cd-loaded conditions. Under Cd-free conditions, aromatic amino acids (phenylalanine, tryptophan and tyrosine) and branched-chain amino acids (leucine, isoleucine and valine) were accumulated more in the leaves of *N*. *praecox* than in *N*. *caerulescens*. Cd stress increased the content of these amino acids in both species but this increase was significant only in *N*. *caerulescens* leaves. Marked differences in the responses of the two species to Cd stress were shown for alanine, phenylalanine, threonine and sarcosine. Cadmium contamination also induced an increase of threonine as alanine and sarcosine decrease, which was larger in *N*. *caerulescens* than in *N*. *praecox*. All these factors contribute to the higher adaptation of *N*. *praecox* to Cd stress.

## Introduction

Cadmium (Cd) is one of the most dangerous heavy metals for nearly all organisms due to its high solubility in water and toxicity even at very low concentrations [1]. Although Cd is toxic to plant growth, it is easily taken up by the roots and translocated to the shoots [2]. Uptake of Cd ions from the soil seems to occur mainly via non-selective transporters of calcium (Ca), iron (Fe), manganese (Mn) and zinc (Zn) [3]. Therefore, Cd may affect nutrient metabolism (uptake, transport and use) and interfere with essential mineral nutrients such as Ca, copper (Cu), Fe, potassium (K), nickel (Ni), magnesium (Mg), Mn and Zn [4–7]. These elements form part of important biomolecules—metalloenzymes and metalloproteins [8]. Copper, Mn, Ni and Zn were found in metalloenzymes as cofactors [8–10]. Metalloenzymes contain an essential metal ion cofactor in their catalytic active sites, forming biological metal complexes that perform a wide range of important functions such as the activation of small molecules, atom transfer chemistry, and the control of oxidation equivalents [8]. Metalloenzymes (superoxide dismutase, urease and cytochrome P450) can also significantly affect the resistance of plants against stress conditions [11, 12].

Many studies have examined the effect of Cd exposure on the physiology and metabolism of plants [1, 2, 6, 7, 13–18]. Cd affects metabolism of amino acids (AA) and organic acids in plants [2, 15]. Amino acids play significant roles in metal binding, antioxidant defence and signalling in plants during heavy metal stress [15, 16]. The metabolism of AA plays an important role in intracellular pH regulation, especially alanine (Ala) and γ-aminobutyrate (GABA) [19–22]. An important role in the growth of plant cell walls and stress adaptation is played by polypeptides and proteins with high contents of proline (Pro), hydroxyproline, glycine (Gly), cysteine, leucine (Leu) and methionine (Met) [23]. Branched-chain AA (BCAA), i.e. Leu, isoleucine (Ile) and valine (Val), play a role during osmotic stress and their accumulation points to the tight regulation of BCAA catabolism by environmental perturbations. Additionally, acetyl-CoA, propionyl-CoA, and acetoacetate, which are the breakdown products of BCAA, are potential energy sources for plants [24]. Phenylalanine (Phe), tyrosine (Tyr) and tryptophan (Trp) are aromatic AA derived from the shikimate pathway and are required for protein synthesis and production of aromatic secondary metabolites, e.g. anthocyanin [25–27], which are important for cell wall extensibility [28]. The AA sarcosine (Sar, N-methylglycine) is an intermediate of trimethylglycine (glycine betaine). Glycine betaine is known to be a metabolite that accumulates during oxidative stress [29]; therefore, Sar declines as a result of the methylation of Sar [30] or due to the lack of Gly caused by the reaction of Gly and choline [31]. Thus, a decline in Sar is related to the biosynthesis of glycine betaine and plant defence against oxidative stress. According to the literature, Sar complexation with metals is one of many functions of these specific AA; however, such complexation has not been confirmed in plants (increase of Sar with increasing toxic Cd content). We assume that one of the functions of Sar is its role as a methyl donor for antioxidative metabolites in plant stress metabolism (decrease of Sar with increasing Cd content).

Free AA have been shown to have functional roles in plant stress tolerance; therefore, our study focused on investigating the changes in the selected free AA of hyperaccumulating plants (*Noccaea caerulescens* and *N*. *praecox*) growing on an environmentally relevant substrate—soil—that occur under chronic stress caused by Cd. The main objective was to characterise changes in AA metabolism that are associated with plant tolerance, and in the contents of selected microelements, in order to relate these changes to the adaptation strategies of *N*. *caerulescens* and *N*. *praecox* growing under Cd stress. The experiment was aimed at AA that are important in: (i) stress responses associated with carbon (C) and nitrogen (N) metabolism (Ala, GABA and ornithine [Orn]); (ii) adaptive responses leading to pH cytosolic regulation (Ala and GABA); (iii) balancing the ratio between different AA pathways (Leu, Ile and Val) to the maintenance of homeostasis; (iv) catabolism of aromatic AA (Phe, Tyr and Trp), which is coupled with the phenylpropanoid pathway and generates antioxidant metabolites; and (v) complexation reactions with Cd and other metals (Sar).

## Material and methods

### Plant material and cultivation conditions

*Noccaea caerulescens* (formerly *Thlaspi caerulescens* J. & C. Presl, F. K. Mey) and *Noccaea praecox* (formerly *Thlaspi praecox* Wulfen, F.K. Mey) are metallophytes that are both able to grow on metal-contaminated soils [32] and accumulate certain trace metals in parts growing above ground [7, 33]. *N*. *praecox* from Mežica, Slovenia and *N*. *caerulescens* from Redlschlag, Austria were used in the pot experiments. Characteristics of the Mežica and Redlschlag sites were published previously [34, 35].

For the cultivation of *Noccaea* plants, 3 kg of soil from the non-polluted site, Prague-Demonstration Field of Czech University of Life Sciences-Výhledy II (see Table 1 for details), was mixed with a nutrient solution consisting of 0.3 g N as NH_4_NO_3_, 0.1 g P and 0.24 g K as K_2_HPO_4_. To the samples to be considered “contaminated”, a Cd solution with a concentration of 90 mg Cd/kg of soil as Cd(NO_3_)_2_·4H_2_O was added. Plants were cultivated in a greenhouse under controlled conditions with a 16 hour day/8 hour night cycle and a temperature cycle of 24°C day/18°C night. The water regime was controlled, and the soil moisture was maintained at 60% maximum water-holding capacity. Each treatment was performed in five replications. Plants were harvested 120 days after Cd application. Samples were kept frozen in liquid N for transport and then at –30°C until extraction.

**Table 1:** Characteristics and total initial Cd concentration in experimental soil.

Soil type/subtype	pH_KCl_	CEC (mmol_(+)_/kg)	C_org_ (%)	Cd_T_ (mg/kg)
Chernozem/modal	7.2 ± 0.1	258 ± 2.8	1.83 ± 0.01	0.42 ± 0.05

CEC, cation exchange capacity; C_org_, organic carbon; Cd_T_, total content of Cd.

### Free amino acid analysis

For the analyses of AA, 0.5 ± 0.05 g samples of fresh biomass were extracted in 10.5 mL of methanol + double distilled H_2_O (7:3, v/v) for 24 hours. Derivatisation of free AA was performed using the EZ:faast set (Phenomenex, USA) following the manufacturer’s instructions. The content of free AA was determined by gas chromatography-mass spectrometry ([GC-MS] Hewlett Packard 6890N/5975 MSD, Agilent Technologies, USA) with a ZB-AAA 10 m x 0.25 mm AA analysis GC column and a constant carrier gas flow (He, 1.1 mL/min). The oven temperature program and MS conditions were the same as described by Pavlík et al. [36].

### Accumulation of cadmium and microelements

Plant samples (0.5 ± 0.05 g dry biomass) were decomposed using the dry ashing procedure. The ash was dissolved in 20 mL of 1.5% HNO_3_ (v/v) (Analytika Ltd., CZ) and the contents of Cd, Cu, Mn, Ni and Zn were determined by inductively coupled plasma optical emission spectroscopy with axial plasma configuration (Varian VistaPro, Varian, Australia). Certified reference material RM NCS DC 73350, poplar leaves (purchased from Analytika, CZ), were mineralised under the same conditions for quality assurance.

### Statistical analysis

The statistical analysis was performed using Statistica 9.0 software (www.statsoft.com). Presented data are the mean values ± standard error (SE). All results were tested by one-way analysis of variance (ANOVA) considering interactions at the 95% significance level (*p* < 0.05) with subsequent Tukey’s honest significant difference (HSD) test and by correlation (*R*^*2*^, *p* < 0.05). A principal component analysis (PCA), in the CANOCO 4.5 program [37], was applied to all collected data as a single set. We used standardisation of species because data of different character were analysed together. PCA was used to make correlations between analysed data and similarity of different treatments visible from the complex data set. The results were visualised in the form of a bi-plot ordination diagram using the CanoDraw program.

## Results

### Effect of Cd on plant biomass and Cd accumulation

As reported in our previous paper [38], Cd induced a greater reduction of yield of above-ground biomass in *N*. *caerulescens* than in *N*. *praecox*. *Noccaea* species showed significant differences of Cd content in plant biomass. The results presented in Table 2 show that *N*. *praecox* accumulated significantly higher Cd content (7045 mg Cd/kg dry weight) than *N*. *caerulescens* (134 mg Cd/kg dry weight). The phytoextraction potential of *Noccaea* species is indicated by the remediation factor (RF), which reflects the amount of metal extracted by plants from the soil during vegetation period. For both plant species, the RF was calculated as follows (1):

$$\text{RF}\;\left(\%\right)=\frac{{\text{Cd}}_{\text{plant}}DM_{\text{plant}}}{{\text{Cd}}_{\text{soil}}w_{\text{soil}}}100$$ (1)

where Cd_plant_ is the content of Cd in plant dry biomass (mg/kg), *DM*_plant_ the dry weight plant biomass yield (kg), Cd_soil_ the total concentration of Cd in the soil (mg/kg) and *w*_soil_ the amount of soil in the pot (kg), modified according to Zárubová et al. [39]. The RF of Cd for *N*. *praecox* and *N*. *caerulescens* was 12.6% and 0.26%, respectively.

**Table 2:** The Cd and microelements content (mg/kg DW) in the above-ground biomass of plants.

Variable	Treatment			
	*N*. *praecox*	*N*. *praecox*-Cd	*N*. *caerulescens*	*N*. *caerulescens*-Cd
**Cd**	26.4^a^^B^ ± 0.7	7045^b^^B^ ± 66	2.4^a^^A^ ± 0.05	134^b^^A^ ± 6
**Zn**	543^a^^A^ ± 46	539^a^^B^ ± 38	495^b^^A^ ± 29	201^a^^A^ ± 16
**Cu**	5.4^a^^A^ ± 0.5	6.0^b^^A^ ± 0.3	6.8^a^^B^ ± 0.7	6.2^a^^A^ ± 0.6
**Mn**	70.1^a^^A^ ± 11.0	76.2^a^^A^ ± 9.2	110.8^a^^B^ ± 8.5	108.4^a^^B^ ± 7.8
**Ni**	71.6^b^^A^ ± 6.7	42.8^a^^B^ ± 3.2	104.5^b^^B^ ± 6.7	9.2^a^^A^ ± 0.9

Treatment abbreviations: *N*. *praecox*, *Noccaea praecox* control plants without cadmium; *N*. *praecox*-Cd, *Noccaea praecox* plants with Cd solution in soil with a concentration of 90 mg Cd/kg of soil; *N*. *caerulescens*, *Noccaea caerulescens* control plants without cadmium; *N*. *caerulescens*-Cd, *Noccaea caerulescens* plants with Cd solution in soil with a concentration of 90 mg Cd/kg of soil.

The values represent the means of data obtained in the experiment (n = 5). Different letters indicate values that are significantly different (*p* < 0.05):

A, B comparison between the two species with the same treatment (growing with or without Cd solution applied to the tested soil)

a, b comparison between treatments in each species.

### Effect of Cd on microelements content

The microelement contents of *N*. *praecox* and *N*. *caerulescens* were significantly different (Table 2). The trends of Ni and Zn changes were similar in both plant species; however, the trends of Cu and Mn changes were opposite. High cation contents were seen in the control treatment of *N*. *caerulescens*. Manganese and Ni contents in the *N*. *caerulescens* control treatment were significantly higher than those in the *N*. *praecox* control treatment (58% and 46% higher, respectively). Cadmium contamination had an effect on the content of some microelements in the above-ground biomass. Cadmium reduced the contents of Zn and Ni but did not significantly alter Cu and Mn contents in any of the treatments. Zinc and Ni contents were significantly affected in Cd-exposed *N*. *caerulescens* (59% Zn reduction; 91% Ni reduction), while Cd-exposed *N*. *praecox* showed reductions of only 1% for Zn and 40% for Ni.

### Effect of Cd on free amino acids content

**Fig 1:**
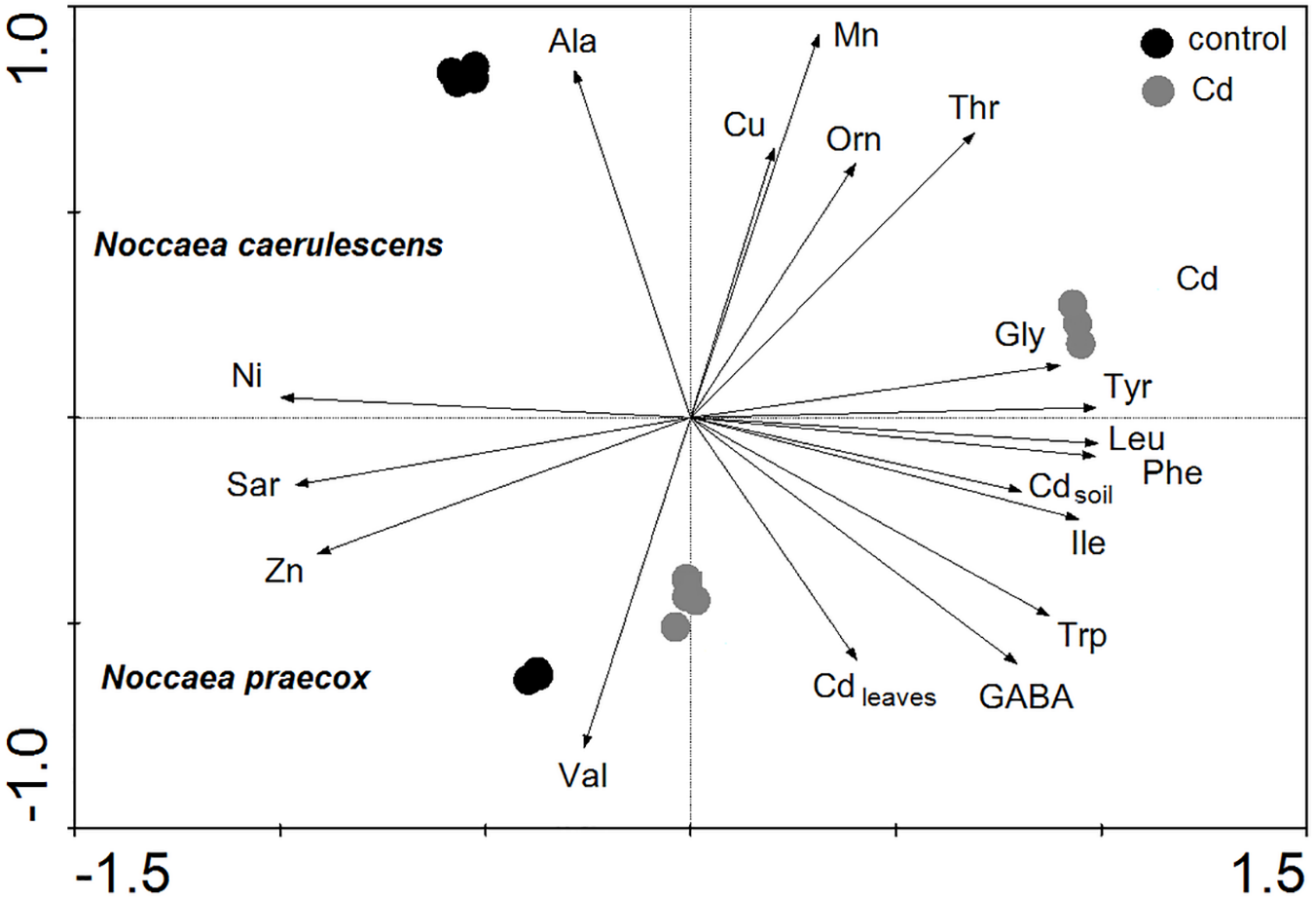
Ordination diagram showing the results of PCA analysis with content of Cd, microelements and selected free amino acids in above-ground biomass of plants. Cd_leaves_, total content of Cd in leaves of plants; Cd_soil_, content of Cd in soil; Cu, Mn, Ni and Zn, total content of elements; Ala, content of alanine; GABA, content of γ-aminobutyrate; Gly, content of glycine; Ile, content of isoleucine; Leu, content of leucine; Orn, content of ornithine; Phe, content of phenylalanine; Sar, content of sarcosine; Thr, content of threonine; Trp, content of tryptophan; Tyr, content of tyrosine and Val, content of valine.

The contents of Ala varied according to species and according to the Cd content in the plants (Table 3 and Fig 1). Decreases of Ala contents were apparent in both plants species growing on the Cd treatments (*N*. *praecox* by 15% and *N*. *caerulescens* by 19% compared to control treatments). Lower Ala contents were confirmed for *N*. *praecox* treatments than in *N*. *caerulescens* treatments. A 22% lower content of Ala was determined in *N*. *praecox* than in *N*. *caerulescens* controls and a 19% lower content in *N*. *praecox*-Cd than *N*. *caerulescens*-Cd.

**Table 3:** The concentrations of selected free amino acids (free AA; μmol/kg FW) in the above-ground biomass of plants.

Free AA	Treatment			
	*N*. *praecox*	*N*. *praecox*-Cd	*N*. *caerulescens*	*N*. *caerulescens*-Cd
**Ala**	772^a^^A^ ± 2	652^b^^A^ ± 65	994^b^^B^ ± 39	805^a^^B^ ± 59
**GABA**	368^a^^A^ ± 6	382^a^^A^ ± 49	333^a^^A^ ± 39	392^a^^A^ ± 54
**Phe**	609^a^^B^ ± 31	625^a^^A^ ± 38	561^a^^A^ ± 39	770^b^^B^ ± 41
**Tyr**	625^a^^A^ ± 71	643^a^^A^ ± 68	599^a^^A^ ± 57	789^b^^B^ ± 39
**Trp**	714^a^^A^ ± 56	747^a^^A^ ± 42	630^a^^A^ ± 46	800^b^^A^ ± 57
**Ile**	137^a^^A^ ± 15	139^a^^A^ ± 11	120^a^^A^ ± 17	168^b^^A^ ± 19
**Leu**	560^a^^A^ ± 34	573^a^^A^ ± 38	537^a^^A^ ± 31	651^b^^A^ ± 41
**Val**	808^a^^B^ ± 75	728^a^^A^ ± 59	601^a^^A^ ± 54	568^a^^A^ ± 50
**Gly**	471^a^^A^ ± 42	446^a^^A^ ± 35	578^b^^B^ ± 41	449^a^^A^ ± 58
**Orn**	180^a^^A^ ± 1	312^b^^A^ ± 23	287^a^^B^ ± 25	336^a^^A^ ± 31
**Thr**	822^a^^A^ ± 9	875^a^^A^ ± 35	1440^a^^B^ ± 50	2348^b^^B^ ± 92

Free amino acid abbreviations: Ala, alanine; GABA, γ-aminobutyric acid; Phe, phenylalanine; Tyr, tyrosine; Trp, tryptophan; Ile, isoleucine; Leu, leucine; Val, valine; Gly, glycine; Orn, ornithine; Thr, threonine.

Treatment abbreviations: *N*. *praecox*, *Noccaea praecox* control plants without cadmium; *N*. *praecox*-Cd, *Noccaea praecox* plants with Cd solution in soil with a concentration of 90 mg Cd/kg of soil; *N*. *caerulescens*, *Noccaea caerulescens* control plants without cadmium; *N*. *caerulescens*-Cd, *Noccaea caerulescens* plants with Cd solution in soil with a concentration of 90 mg Cd/kg of soil.

The values represent the means of data obtained in the experiment (n = 5). Different letters indicate values that are significantly different (*p* < 0.05):

A, B comparison between the two species with the same treatment (growing with or without Cd solution applied to the tested soil)

a, b comparison between treatments in each species.

GABA contents typically increase in response to various stresses; however, our results showed no significant differences in the GABA content between controls and Cd treatments or between species. The relationship between the contents of GABA and Ala in the above-ground biomass of plants was confirmed using linear correlation. A significant negative relationship was calculated for both *N*. *praecox* and *N*. *caerulescens* plants (*R*^*2*^ = 0.94); thus, GABA increased when the Ala content decreased.

The results indicate that two groups of AA: aromatic AA (such as Phe, Tyr and Trp, which are necessary for protein biosynthesis, for the biosynthesis of auxin from Trp, and for generation of antioxidant metabolites from Phe and Tyr); and branched-chain AA (such as Ile, Leu, and Val, which are the building blocks of proteins), accumulated more in the leaves of the control *N*. *praecox* treatment than in the leaves of the control *N*. *caerulescens* treatment (by 8% and 16%, respectively). Our analyses revealed that Cd stress increased the contents of these AA in both species, but this increase was significant only in Cd- exposed *N*. *caerulescens* (*N*. *caerulescens*-Cd, Table 3).

Phenylalanine was present in the two species in significantly different concentrations. Cadmium contamination significantly affected its content in *N*. *caerulescens*-Cd (increased by 37%) but had little effect on the phenylalanine content in Cd-exposed *N*. *praecox* (*N*. *praecox*-Cd, increased by 3%). The contents of Tyr and Trp were increased through Cd treatment, but a significant increase of Trp was only observed for *N*. *caerulescens*-Cd (by 27%). There was a strong correlation between the Cd content in plants and the content of these AA (*R*^*2*^ = 0.86 for Phe, 0.81 for Tyr, and 0.77 for Trp).

**Fig 2:**
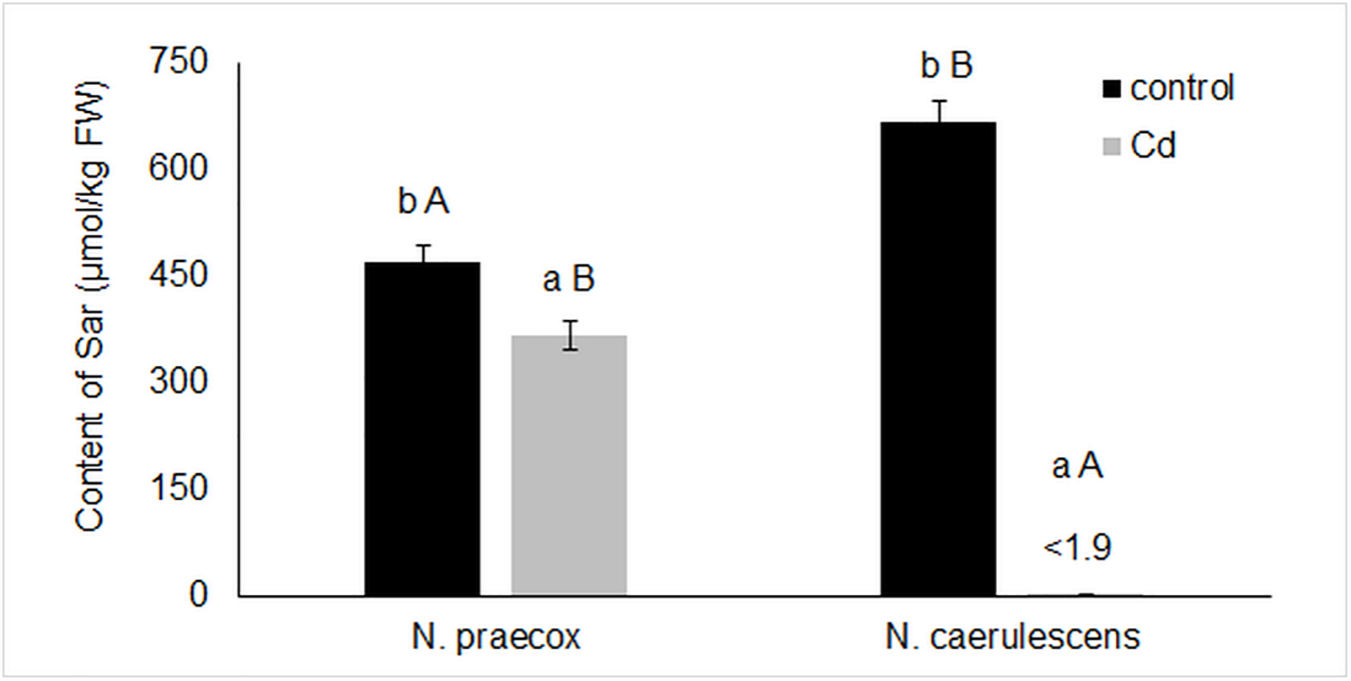
The content of Sar (μmol/kg FW) in the above-ground biomass of plants. Limit of detection: 1.9 μmol/kg. The values represent the means of data obtained in the experiment (n = 5). Different letters indicate values that are significantly different (*p* < 0.05): A, B comparison between the two species with the same treatment (growing with or without Cd solution applied to the tested soil); a, b comparison between treatments in each species.

Results in Table 4 show that Phe, Trp, Tyr, Leu, Ile and Val correlated with Sar (*R*^*2*^ = 0.42–0.70). Sarcosine was only found in *N*. *praecox*, *N*. *praecox*-Cd and *N*. *caerulescens*; Sar was below the detection limit in *N*. *caerulescens*-Cd (Fig 2). As shown by the results in Table 4, Sar correlated with nitrogen-transport AA, i.e. aspartate (Asp), and also with AA in different metabolic pathways such as Ala, Gly, Trp, threonine (Thr) and Ile. The results in Table 4 confirm the relationships of these AA shown in the ordination diagram from the PCA analysis (Fig 1).

**Table 4:** Correlation (*R*^*2*^) of selected free amino acids in the above-ground biomass of plants.

Variable	Ala	GABA	Phe	Tyr	Trp	Ile	Leu	Val	Gly	Orn	Thr	Sar	Asp
**Ala**	x	0.94	0.62	0.61	0.59	0.26	0.80	0.34	0.03	0.05	0.06	0.54	0.34
**GABA**	0.43	x	0.82	0.81	0.76	0.66	0.80	0.13	0.55	0.04	0.27	0.33	0.13
**Phe**	0.25	0.99	x	0.99	0.61	0.78	0.80	0.17	0.94	0.17	0.92	0.42	0.21
**Tyr**	0.41	0.97	0.99	x	0.87	0.81	0.80	0.23	0.96	0.17	0.88	0.56	0.32
**Trp**	0.70	0.41	0.86	0.87	x	0.84	0.80	0.67	0.54	0.28	0.60	0.46	0.14
**Ile**	0.80	0.16	0.64	0.66	0.84	x	0.80	0.61	0.88	0.22	0.82	0.70	0.27
**Leu**	0.51	0.95	0.99	0.86	0.63	0.97	x	0.23	0.96	0.14	0.79	0.60	0.33
**Val**	0.91	0.16	0.24	0.12	0.17	0.17	0.29	x	0.01	0.94	0.30	0.61	0.54
**Gly**	0.46	0.59	0.77	0.82	0.50	0.84	0.80	0.75	x	0.36	0.84	0.57	0.32
**Orn**	0.29	0.92	0.92	0.94	0.39	0.23	0.80	0.76	0.11	x	0.32	0.13	0.20
**Thr**	0.05	0.79	0.90	0.87	0.72	0.75	0.78	0.17	0.83	0.33	x	0.26	0.19
**Sar**	0.64	0.27	0.02	0.61	0.66	0.06	0.06	0.21	0.52	0.22	0.58	x	0.83
**Asp**	0.37	0.13	0.10	0.11	0.35	0.73	0.11	0.04	0.91	0.08	0.61	0.92	x

Free amino acid abbreviations: Ala, alanine; GABA, γ-aminobutyric acid; Phe, phenylalanine; Tyr, tyrosine; Trp, tryptophan; Ile, isoleucine; Leu, leucine; Val, valine; Gly, glycine; Orn, ornithine; Thr, threonine; Sar, sarcosine; Asp, aspartate.

The difference in Gly content between the control treatments of both species was not statistically significant, but the content in *N*. *caerulescens* was higher (by 18%). A significant decrease in Gly was found for *N*. *caerulescens*-Cd relative to the control (22%). A not significant Cd-induced decrease of Gly was shown for *N*. *praecox* (6%). Significant differences were determined between the tested species for Orn (59% greater in *N*. *caerulescens* than in *N*. *praecox*) and between control treatment of *N*. *praecox* and *N*. *praecox*-Cd (73% increase).

Increases of free Leu and Ile concentrations under Cd stress were only observed for *N*. *caerulescens* (21% increase for Leu, and 40% increase for Ile). No significant changes in these AA were seen for *N*. *praecox* treatments. A different trend was observed for Val in both Cd treatments, but the changes were not significant. The correlations between Cd contents in plants and Leu and Ile contents were apparent (*R*^*2*^ = 0.65–0.67); however, this relationship was not confirmed for Val (*R*^*2*^ = 0.17). Threonine and Met serve as substrates for synthesis of Ile and their syntheses and catabolism also affect the availability of Ile. A significant correlation was found between Thr and Ile (*R*^*2*^ = 0.75); however, the Met concentrations were below the detection limit of GC-MS, so such a correlation could not be established. While both plant species tended to have higher concentrations of Thr in the Cd treatments, the difference between the Thr contents of the different species was significant. A significant increase in Thr was only observed in *N*. *caerulescens*-Cd, which increased by 63% relative to the control *N*. *caerulescens* plants.

### Results of principal component analysis

The first axis of the PCA analysis explained 59%, the first two axes 86%, and the first four axes together 99.9% of the variability of all analysed data (Fig 1). The length and direction of the vectors indicate the strength of the vector effect and correlation between vectors. Long vectors for all our parameters indicate that the vector greatly affected the results of the analysis. Aromatic AA (Phe, Tyr and Trp) and BCAA (Ile, Leu and Val) were positively correlated with the content of Cd, as indicated by the angles smaller than 90° between the vectors for these parameters. On the other hand, Ala was clearly negatively related to the content of Cd. The first ordination axis divided individual pots into the *N*. *caerulescens* group on the top-side and *N*. *praecox* on the bottom-side of the diagram, indicating a large effect of plant species on the content of microelements and selected free AA. For *N*. *caerulescens*, marks for the control and Cd-exposed treatments were located in different parts of the diagram, which indicates a large effect of treatments on all the recorded data. In contrast, the data for *N*. *praecox* showed a minimal effect of treatments.

## Discussion

### Different accumulation of microelements in *Noccaea* plants is related to toxic Cd effect

*Noccaea* plants originating from heavy metal-contaminated regions are known to be metallicolous [40]. The two species under study differ depending on their adaptation to different soil contaminants. The *N*. *caerulescens* species from Redlschlag, Austria has adapted to growth on serpentinite (soil naturally rich in Ni, Co and Cr). This is in contrast to *N*. *praecox*, which originated from Mežica, Slovenia and has adapted to growth on soil with high Cd, lead and Zn contamination. *N*. *caerulescens* from serpentine soil containing an excess of Mg has been shown to adapt to different environments by increasing the uptake of cations and allocating more energy to cation absorption [32]. For this reason, Cu, Mn and Ni accumulation were significantly higher in the *N*. *caerulescens* control treatment compared to the *N*. *praecox* control treatment. The tested *Noccaea* plants also exhibited differences in the extent to which they reduced the concentration of various elements. Plants exposed to Cd showed decreased concentrations of Zn and Ni and the decrease in both these elements was significantly stronger in *N*. *caerulescens*-Cd than in *N*. *praecox*-Cd. These results confirmed the higher metal accumulation and tolerance in metallicolous *N*. *praecox* in contrast to serpentinite-grown *N*. *caerulescens* [17, 40, 41].

Based on the above facts we affirm that *N*. *praecox* plants are more efficient in their use of the accumulated cations which are important cofactors for metalloenzymes. The biogenic elements Zn, Cu, Mn, Fe and Ni are cofactors of metalloenzymes such as superoxide dismutase or cytochrome P450, a broad class of important biomolecules that form biological complexes with metals that perform a wide range of important functions including defence against oxidative stress [42]. Another metalloenzyme, Ni-urease, affects the regulation of N and C metabolism [43] by linking the urease cycle to the tricarboxylic acid (TCA) cycle. Regulation of the ratio of N to C is very important for overcoming environmental or oxidative stress. The formation of a bond between metals and enzymes is dependent on the metal concentration in the cell [44]. A decline of metal contents may thus result in a decrease in metalloenzyme activities. We propose that the increased uptake of Cu, Mn and Ni by *N*. *caerulescens* control treatment plants in contrast to *N*. *praecox* control treatment plants is the result of selection pressure from the serpentine area. The decrease in the uptake of some elements by *N*. *caerulescens*-Cd plants from Cd-contaminated soil in contrast to *N*. *praecox*-Cd is related to the more efficient use of these elements in metabolism. Metalloenzymes are therefore important for overcoming adverse conditions causing oxidative stress, which damages plant growth and development.

### Difference in alanine and γ-aminobutyrate accumulation is related to pH regulation

It has been reported by several authors that Ala is markedly accumulated in response to various stresses and its role is discussed especially in relation to intracellular pH regulation [21, 45, 46]. Our results, however, demonstrate an opposite trend; Ala contents were decreased in both species growing on Cd-contaminated soil. Similar results were reported for non-hyperaccumulating plants growing on slightly arsenic (As)-contaminated soil [47] and for tobacco growing on Zn-contaminated soil [16]. The increased content of free Ala might be caused by a reduction in the rate of protein synthesis and an increased Ala synthesis due to disturbance of alanine aminotransferase reactions [48]. Our results showed that hyperaccumulating plants do not accumulate Ala in the cytosol as part of pH regulation, but they can use it for the biosynthesis of proline/alanine-rich protein kinases [49] or histidine- and alanine-rich proteins [50].

An increase in levels of GABA at the expense of Ala leads to a higher increased pH in the cytosol, which in turn alters the activities of enzymes as well as the activity and transport of plant hormones. We suggest that GABA may be more significant for effective pH regulation in the cytosol of both *Noccaea* species than Ala [51], this is also relevant when considering the isoelectric points of the two AA. One route for GABA synthesis is non-enzymatic conversion of Pro to GABA, which was recently reported to be an alternative route for providing adenosine triphosphate [52], to C and N under conditions of oxidative stress [53]. GABA plays different roles in plant metabolism, including N-C metabolism, energy balance, signalling and development, and stress defence [51]. The GABA shunt plays a key role in controlling the N/C ratio by linking AA metabolism and the TCA cycle, which is essential for higher plant species [22]. This role is confirmed by our finding of a significant correlation between GABA content and the total content of free AA (*R*^*2*^ = 0.99). Increased GABA levels in response to short exposures to different abiotic and biotic environmental stressors are commonly observed in several plants [20, 54]. A significant increase of GABA under Cd stress has been observed in tomato plants [6] while in our hyperaccumulator species GABA accumulation was not significant. This indicates that tomato plants are less well adapted to the toxic effect of Cd than *Noccaea* species.

### Difference in accumulation of aromatic amino acids is related to antioxidative metabolism

Under Cd stress, the levels of Phe, Tyr and Trp were significantly increased only in *N*. *caerulescens* plants, which are less Cd-tolerant than *N*. *praecox*. These AA are necessary not only for protein biosynthesis but also for other important processes. For instance, Phe is also a substrate for the phenylpropanoid pathway, which produces a wide range of plant secondary products, especially antioxidative metabolites (flavonoids, anthocyanins, lignins and phenylpropanoic acids including salicylic acid) and phenolic compounds [55] that may act as growth promoters or growth inhibitors. The significant increase of Phe in *N*. *caerulescens*-Cd suggests a higher production of antioxidative metabolites. Tryptophan plays a major role in the regulation of plant development and defence responses [56] and is required for the biosynthesis of auxin (indole-3-acetic acid), a phytohormone that is necessary for the growth and development of plant cells [36], and plays an important role in the adaptation to environmental stress [57]. Exogenous Trp can be effectively used to improve plant growth in soil contaminated with Cd [56]. Biosynthesis of Trp is induced by stresses; an increased content of Trp has thus been found in plants under drought [58] and Cd stress [59]. Bivalent Trp side chains and metal ions were found to mutually interact [60]; this finding corresponds with our results. A significant increase in Trp was reported only in *N*. *caerulescens*-Cd plants, rather than in the more Cd-tolerant *N*. *praecox* plants.

### Difference in glycine, sarcosine and ornithine accumulation is related to tolerance of studied hyperaccumulators

The presence and usability of AA (such as α-aminoadipic acid and Sar) occurring infrequently and/or at very low levels in plant stress metabolism, can be explained by epigenetic changes induced by stress in the tested *Noccaea* species [61].

The difference in Gly content between control treatments of both species was not significant. A significant decrease in Gly levels was only found for *N*. *caerulescens*-Cd. Glycine is a crucial amino acid for the biosynthesis of Sar and Cys via Ser. Glycine is involved in the biosynthesis of phytochelatines and antioxidant metabolites, and is also found in glycine-rich proteins that affect the growth and function of cell walls. Therefore, the decreased Gly contents in both species must be seen as the activation of adaptation processes to the toxic effect of Cd, as was the case with lettuce exposed to dust containing As and Cr [36]. Asp is decreased in less tolerant *N*. *caerulescens* as in lettuce [36], but is increased in tolerant *N*. *praecox*. Aspartate is an amino donor for the biosynthesis of Gly, as confirmed through the correlation of these AA (Table 4 and S1 Fig).

The results showed no correlation between Met and Sar because the contents of Met were below the detection limit. It is generally known that Met participates in the N-methylation of Gly via S-adenosylmethionine by acting as a methyl donor [20, 62–64]. Oxidative stress of *N*. *caerulescens*-Cd was significantly stronger than that of *N*. *praecox*-Cd, as shown by a dramatic decrease of Sar content in *N*. *caerulescens*-Cd. In the Cd-treated plants Sar was only detected in *N*. *praecox*-Cd (the content of Sar in *N*. *caerulescens*-Cd was below the detection limit). We assume that the high Cd accumulation in *N*. *praecox*-Cd could be due to the chelation of Cd by Sar, because Sar can form complexes with Cd and other metals [65, 66] and can protect nucleic acids from oxidative stress [67]. This is in contrast with the fact that these chelates and free AA have a positive relationship with the toxic elements, such as with the chelation of Zn and histidine [67]. Sar-Ni complexes are probably degraded, as indicated by the decrease of Ni and Sar contents under Cd stress. The slow decline of Sar levels can be an advantage for *N*. *praecox* relative to *N*. *caerulescens* if the primary importance of Sar in studied hyperaccumulating plants is not in the formation of chelates with toxic Cd. The effect of Sar on changes of Cd content in plants was confirmed by the positive correlation coefficient (*R*^*2*^ = 0.52). A correlation coefficient of *R*^*2*^ = 0.29 was calculated for the effect of Cd contents on Sar. Based on these results we can speculate that complex of Sar and Cd is formed only under the effect of increased Cd content in tested plants. As is clear from our results, Sar chelates probably have only a secondary meaning for detoxification of Cd.

In *Noccaea* species, as shown in the diagram of Sar metabolism and the related AA (S1 Fig), Sar is used in metabolic pathways of other AA. Catabolism of 5-methyl cytosine to cytosine (a step in DNA methylation) is characterised as an epigenetic change [61]. In plants, this effect is related to an increase of methyl donors for biosynthesis of specific antioxidant metabolites [63, 68]. The catabolism of Sar therefore produces Gly and a methyl donor, which is transformed to homocysteine via formation of Met. Our results indicate a Gly decrease that is similar to our previous results [38] and is caused by Gly incorporation into serine, which is significantly enhanced in hyperaccumulating *Noccaea* species. The decrease of Sar is consistent with its role as a methyl donor for formation of specialised compounds, e.g. antioxidant metabolites [26, 27] which arise from AA, e.g. from Phe and Tyr. A significant correlation was therefore observed between the content of Gly and Sar including aromatic AA and mutual correlations of Sar and Tyr, which are the main substrates for the formation of antioxidant secondary metabolites such as polyamines [63], tocopherols [69, 70] phenylpropanoid metabolites including flavonoids, stilbenes etc. [62, 68, 71]. This is confirmed by the results of PCA (Fig 1) which imply that the content of Sar is inversely proportional to the content of Tyr and Gly. Simultaneously, Cd, which causes oxidative stress, is an important factor for production of Sar which is significantly connected with the content of Ni (Fig 1). Our results thus show that Sar content could be a significant factor (methyl donor) for the adaptability of the hyperaccumulators to oxidative stress, and the differences in adaptability between species which are directly proportional to the RF. Activation of the transmethylation cycle involves activation of O-, C- and/or N-methyltransferase and O-, C- and/or N-demethylase [62, 63, 68]. Based on these findings and with indirect evidence provided in this study we can infer that the tested hyperaccumulators, form methyl pools not only by choline and betaine [72, 73], but also by Sar, whose content decreases with increased Cd content and which originates from the metabolism of choline, betaine, N-dimethylglycine and glycine [30]. Sar catabolism to Gly then yields the methyl group used, via the transmethylation cycle of stress metabolism, for the biosynthesis of the above antioxidant compounds.

The activities of plant desaturases can be affected by Ni content. The effect of Ni on nitrogen metabolism in plants was described previously [43] and these results are supported by those reported here, which show a significant effect of decreased Ni on the AA levels. High plant Ni content is linked to the maximum efficiency of Ni incorporation into the active centre of ureases and glyoxalases. Ureases and glyoxalases play a significant role in cell catabolism [43, 74] and AA catabolism which are associated with plant senescence. The glyoxalase pathway and the two enzymes (glyoxalase I and glyoxalase II) have a significant effect on plant stress metabolism [12, 75]. Ureases are metalloenzymes that catalyse the hydrolysis of urea, an intermediate of plant arginine catabolism that is involved in the remobilisation of nitrogen from tissues [76]. Arginine degradation in the mitochondria via arginase activity leads to the formation of Orn and urea (urease-ornithine cycle). Ornithine can be used in glutamate synthesis, and urea is a source of N that can be sensed by plant cells and used for AA synthesis [77]. An unrelated study showed how urea and Orn accumulated in *Arabidopsis* plants, when Ni was a limiting factor for urease activity. Our results showed an increase of Orn in both *N*. *praecox*-Cd and *N*. *caerulescens*-Cd treatments; these increased Orn contents in both species may be related to decreased Ni contents in *N*. *caerulescens*-Cd and *N*. *praecox*-Cd. Ornithine contents are associated with N and C metabolism through a transfer of an AA, Asp, and with the TCA cycle via fumarate. An alternative pathway for AA regulation via Orn may be characteristic for these hyperaccumulators [78]. Nitrogen obtained from the urease-ornithine cycle may be used for the production of AA studied here, e.g. Ala, Sar and Val.

Urease and glyoxalase activity can be a source of energy during stress metabolism in hyperaccumulating plants [75]. For the above reasons, the use of Ni as a cofactor of urease and glyoxalases is important for the adaptation of hyperaccumulators to Cd contamination. A higher Ni content in *N*. *praecox*-Cd than in *N*. *caerulescens*-Cd was found in our experiments. This finding may explain the better ability of *N*. *praecox* from Mežica to use metabolite catabolism for the formation of compounds involved in the detoxification of toxic elements.

### Difference in accumulation of branched-chain amino acids is related to amino acid homeostasis

Amino acid homeostasis is essential for growth, development and defence of plants against stress [79]. This homeostasis is regulated by *de novo* biosynthesis, uptake/translocation, and protein synthesis/degradation [80]. Leucine, Ile and Val catabolism play physiological roles beyond maintaining AA homeostasis [81]. These AA are pivotal in balancing the fluxes between different AA pathways [80]. The accumulation of Leu, Ile and Val may serve to promote stress-induced protein synthesis and may act as signalling molecules to regulate gene expression [24]. Only Leu and Ile showed significant increases for the *N*. *caerulescens*-Cd treatment. This finding confirmed that *N*. *caerulescens* plants are less adaptable to Cd stress and showed that in *N*. *caerulescens* plants stress activated enzymes associated with degradation proteins and senescence.

Leucine, together with Zn, is present in many enzymes that degrade proteins, such as leucine aminopeptidases [82]. These enzymes are involved in defence against stress and are connected to the transport of auxin, the formation of jasmonic acid and plant senescence induced by stress such as that caused by heavy metals. A higher content of Leu was determined in the Cd treatment of *N*. *caerulescens* plants compared to *N*. *praecox* plants. Our findings may be connected to the significantly lower contents of Zn (cofactor of superoxide dismutase) in *N*. *caerulescens* than in *N*. *praecox*. The reduction of Zn in *N*. *caerulescens* plants compared with *N*. *praecox* is one of the reasons for reducing the capacity of the plant antioxidant system [11, 42].

Plant pathways regulating Thr, Met and Ile metabolism are highly interconnected. Synthesis of Ile proceeds through Thr and Met; therefore, their synthesis and catabolism at different developmental plant phases and under different environmental conditions also influences the availability of Ile [24].

Differences in adaptability of the hyperaccumulators result not only from the regulation of metabolism of the studied AA but also the regulation of other AA [38] and fatty acids [17, 18]. The high adaptability of *N*. *praecox* to the toxic effect of Cd is the result of a complex regulation of AA and fatty acids metabolism.

## Conclusion

Our results emphasise that *N*. *praecox* and *N*. *caerulescens* growing under the selective pressure of differently contaminated locations can differ in physiological performance. The results point to complex changes in the regulation of stress metabolism of plants exposed to selection pressure during their phylogenetic development in the specific polluted environment. *Noccaea* species showed contrasting responses to Cd contamination in the content of microelements and AA metabolism. Manganese and Ni contents in the sample of the *N*. *caerulescens* control treatment were significantly higher than in the *N*. *praecox* control treatment. The ability of *N*. *caerulescens* to take up cations is a result of the selective pressure of growth in a contaminated area. The contents of microelements in leaves of both *Noccaea* species were affected by Cd supply. Cadmium reduced Zn and Ni contents mainly in the *N*. *caerulescens*-Cd treatment, but did not significantly affect Cu and Mn in any of the treatments. Different responses to Cd contamination of *Noccaea* species were found for Ala, Phe, Thr and Sar. The more significant changes of contents of Thr (increase) and Sar (decrease) were determined from responses in *N*. *caerulescens* rather than in *N*. *praecox* under Cd stress. Plant growth is limited by these changes resulting from stress metabolism. The data on all tested parameters confirmed the higher adaption of *N*. *praecox* than *N*. *caerulescens* to Cd-induced stress.

## Supporting information

S1 Fig The significance of sarcosine in the non-stressed and stressed metabolism of hyperaccumulating plants. Red colour, amino acids and their analogues. Blue colour, methyl and major metabolites, which are donors and/or acceptors of methyl. Bold font, metabolites, which are commented or mentioned in results and discussion. AAAs, aromatic amino acids; ALA, alanine; ASP, aspartic acid; Betaine, glycine betaine; CYSTA, cystathione; CYS, cysteine; Gly, glycine; HCY, homocysteine; HSE, homoserine; Me, methyl; MET, methionine; PHE, phenylalanine; SAHCY, S-adenosylhomocysteine; SAM, S-adenosylmethionine; SAR, sarcosine; SER, serine; TCA cycle, tricarboxylic cycle (citrate cycle); TRP, tryptophan; TYR, tyrosine; 5MeCytosine, 5-methylcytosine.
(TIF)
