# Supplementary figures and images for: Cadmium toxicity induced contrasting patterns of concentrations of free sarcosine, specific amino acids and selected microelements in two Noccaea species
Source: PLoS One. 2017 May 19;12(5):e0177963. doi: 10.1371/journal.pone.0177963 (PMC5438182; doi:10.1371/journal.pone.0177963)

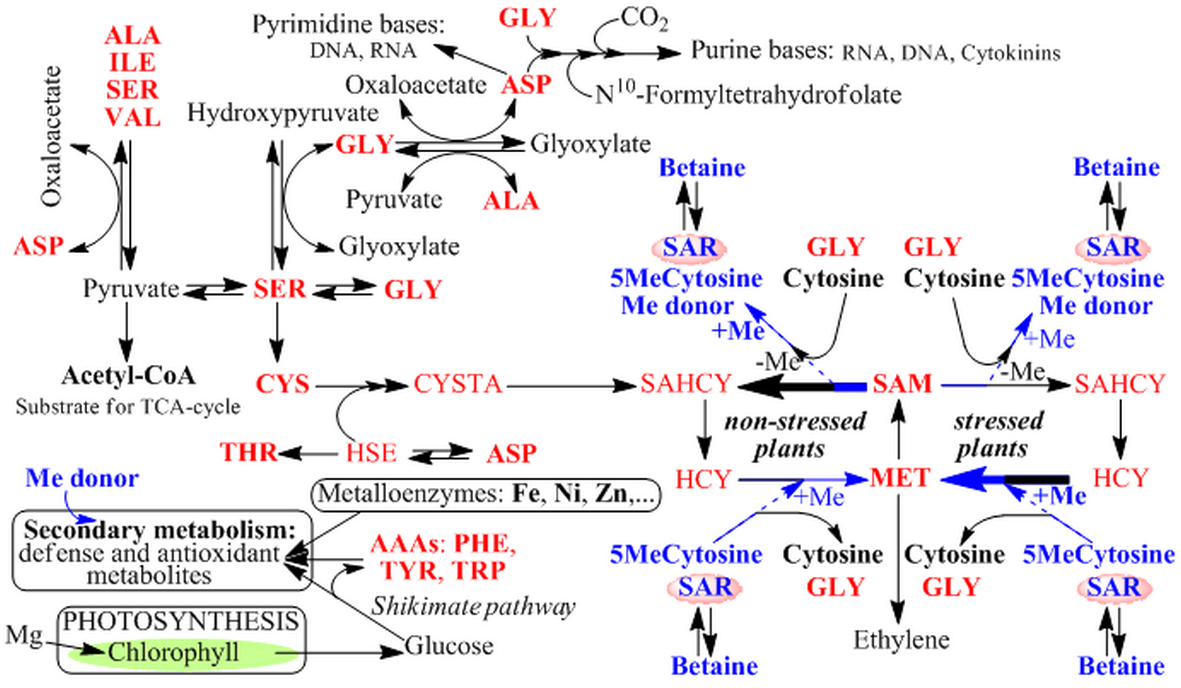

Supplement: S1 Fig — Red colour, amino acids and their analogues. Blue colour, methyl and major metabolites, which are donors and/or acceptors of methyl. Bold font, metabolites, which are commented or mentioned in results and discussion. AAAs, aromatic amino acids; ALA, alanine; ASP, aspartic acid; Betaine, glycine betaine; CYSTA, cystathione; CYS, cysteine; Gly, glycine; HCY, homocysteine; HSE, homoserine; Me, methyl; MET, methionine; PHE, phenylalanine; SAHCY, S-adenosylhomocysteine; SAM, S-adenosylmethionine; SAR, sarcosine; SER, serine; TCA cycle, tricarboxylic cycle (citrate cycle); TRP, tryptophan; TYR, tyrosine; 5MeCytosine, 5-methylcytosine. (TIF) [file pone.0177963.s001.tif]
